# Tissue‐engineered tendon nano‐constructs for repair of chronic rotator cuff tears in large animal models

**DOI:** 10.1002/btm2.10376

**Published:** 2022-07-22

**Authors:** Yonghyun Gwon, Woochan Kim, Sunho Park, Yang‐Kyung Kim, Hyoseong Kim, Myung‐Sun Kim, Jangho Kim

**Affiliations:** ^1^ Department of Convergence Biosystems Engineering Chonnam National University Gwangju Republic of Korea; ^2^ Department of Rural and Biosystems Engineering Chonnam National University Gwangju Republic of Korea; ^3^ Interdisciplinary Program in IT‐Bio Convergence System Chonnam National University Gwangju Republic of Korea; ^4^ Department of Physical and Rehabilitation Medicine Chonnam National University Medical School & Hospital Gwangju Republic of Korea; ^5^ Department of Orthopaedic Surgery, Chonnam National University Medical School & Hospital Gwangju Republic of Korea; ^6^ Institute of Nano‐Stem Cells Therapeutics, NANOBIOSYSTEM Co., Ltd Gwangju Republic of Korea

**Keywords:** chronic rotator cuff tears, large animal model, tendon nano‐construct, tendon regeneration, tendon‐to‐bone interface

## Abstract

Chronic rotator cuff tears (RCTs) are one of the most common injuries of shoulder pain. Despite the recent advances in surgical techniques and improved clinical outcomes of arthroscopically repaired rotator cuffs (RCs), complete functional recovery—without retear—of the RC tendon through tendon‐to‐bone interface (TBI) regeneration remains a key clinical goal to be achieved. Inspired by the highly organized nanostructured extracellular matrix in RC tendon tissue, we propose herein a tissue‐engineered tendon nano‐construct (TNC) for RC tendon regeneration. When compared with two currently used strategies (i.e., transosseous sutures and stem cell injections), our nano‐construct facilitated more significant healing of all parts of the TBI (i.e., tendon, fibrocartilages, and bone) in both rabbit and pig RCT models owing to its enhancements in cell proliferation and differentiation, protein expression, and growth factor secretion. Overall, our findings demonstrate the high potential of this transplantable tendon nano‐construct for clinical repair of chronic RCTs.

## 1 INTRODUCTION

Rotator cuff tears (RCTs), one of the most common forms of shoulder injury, cause substantial pain and shoulder dysfunction.^1^ In the early stages of injury, a significant number of such tears are asymptomatic^2^, ^3^; however, these can progress to a degenerative disease and then further to a chronic tear, with a prevalence of over 50% in patients of 50 years of age or older.^2^, ^4^ One of the main clinical treatment methods is arthroscopic rotator cuff (RC) surgery, which is commonly performed worldwide.^5^, ^6^, ^7^, ^8^ However, despite recent advances in surgical techniques and improved clinical outcomes for arthroscopically repaired RCTs, perfect structural recovery of the tendon‐to‐bone interface (TBI) after arthroscopic repair of large and massive RCTs is still unattainable.^7^, ^9^ The TBI of the RC is a gradient structure consisting of four regions: the tendon region, which is composed of well‐aligned collagen I fibers; an uncalcified fibrocartilage zone; a calcified fibrocartilage zone; and the bone region, which is composed of collagen type I.^10^ The regions forming this gradient structure are continuous, with no clearly defined borders in between, and they are not clearly distinguishable even at the ultrastructural level.^11^ This unique structure is responsible for distributing mechanical stress and enhancing the bonding strength between soft and hard tissues. Because of this structure, full functional recovery of the RC cannot be achieved without regeneration of the TBI. In fact, incomplete TBI regeneration after arthroscopic repair is responsible for the high retear rates of up to 27%–94%.^2^ Therefore, a new strategy is needed to aid complete TBI regeneration.

Biological scaffolds have emerged as a promising alternative to current arthroscopic repair. Currently, collagen‐based biological scaffolds (e.g., GRAFTJACKET™ by Geistlich) are most commonly used for the clinical repair of RCTs. This type of scaffold contains extracellular matrix (ECM) structures typically extracted from sources such as the porcine intestinal submucosa, porcine dermis, human fascia, and human dermis. Although collagen is a biomaterial that clinical physicians worldwide are most familiar with, collagen‐based biological scaffolds have not shown promising clinical outcomes for RCT repair owing to their low functionality, high swelling rate, bioresorbability, low mechanical support of the RC tendon, and high costs over the past few years.^12^, ^13^, ^14^, ^15^ To overcome these limitations, researchers have fabricated biomimetic scaffolds based on the synthetic biodegradable polymers, such as polycaprolactone (PCL), poly(lactic‐*co*‐glycolic acid), and polylactic acid, to mimic the ECM of the RC tendon as an alternative strategy for tendon tissue regeneration.^16^, ^17^, ^18^, ^19^ Peach et al. reported the regeneration effect of an electrospun PCL scaffold that mimicked the RC tendon tissue microenvironment in a rat model.^20^ Inspired by the high alignment and well‐defined organization of the ECM of natural tendons, our group also developed a PCL‐based nanotopographic scaffold for RC tendon regeneration.^19^ However, despite rapid advances in the development of biodegradable biomimetic scaffolds, the clinical efficacy of a scaffold‐only strategy for RC tendon regeneration is still questionable, as many in vivo studies have not shown a significant effect with this method. Furthermore, to the best of our knowledge, there are very few clinical trials and no reports on the clinical efficacy of biodegradable biomimetic scaffolds.

Recently, stem cell‐based strategy has received tremendous attention for promising candidates for RCT regeneration.^21^, ^22^, ^23^, ^24^ The key factors of which are the regeneration of the TBI through the direct differentiation of stem cells into bone, tendon, cartilage, and ligament (i.e., the structures of the TBI) and the autocrine and paracrine effects of growth factors and cytokines from the stem cells. Kim et al. demonstrated that the application of adipose‐derived mesenchymal stem cells (MSCs) after surgical repair of an RCT could improve the shoulder functions.^22^ In a first‐ever human clinical trial, Jo et al. injected adipose‐derived MSCs into the RC of patients; although the therapy improved the shoulder functions slightly and relieved pain, it did not directly promote RC regeneration.^25^ Despite the slightly positive clinical outcomes, stem cell‐based therapy still has the following critical limitations: (i) the efficiency of stem cell transplantation into the injury site is very low (i.e., the loss rate due to injection is high) and (ii) the injected stem cells fail to achieve stable adhesion and continuous proliferation in the RC tendon, resulting in limited success in repair of the tear.^26^, ^27^, ^28^ Therefore, there is a need for a functional platform or construct that enables integration of the transplanted stem cells into the tissues and provides an environment that promotes continuous tissue regeneration.

As described earlier, the TBI of RCs consists of a structural and compositional gradient integrated through fibrocartilaginous junctions.^29^, ^30^ Because of its hierarchical and complex structure, functional regeneration of the TBI is restricted and thus considered a great clinical challenge. An ideal construct for RCT repair should provide an environment conducive to overcoming the restricted healing capacity of the TBI. The transplantable construct should also exhibit sufficient mechanical properties for supporting the RC tendon as well as allow for the control of biodegradability and cell functions to enable functional TBI and thereby RC tendon regeneration.^31^, ^32^ With these key considerations in mind, we propose herein a novel tissue‐engineered tendon nano‐construct (TNC) composed of human mesenchymal stem cells (hMSCs) on a scaffold that mimics the highly aligned nanotopographic structures of the ECM of native tendon tissue. Using capillary force lithography, we first fabricated a US Food and Drug Administration‐approved PCL‐based biomimetic scaffold bearing a nanotopographic surface possessing highly aligned ECM‐like structures. The TNC was then developed by incorporating hMSCs onto the fabricated scaffold. Next, we investigated the influence of the nanotopographic cues on the hMSCs effects on RC tendon regeneration in rabbit and pig RCT models. To verify the in vivo regeneration effects of the TNC, we also conducted in vitro experiments to investigate the influence of the scaffold's nanotopographic cues on the morphology, adhesion, proliferation, and mineralization of the hMSCs as well as the expression of proteins related to tenogenic differentiation and tendon regeneration and secretion of growth factors by these cells.

## 2 RESULTS AND DISCUSSION

### 2.1 Characteristics and transplantation of tissue‐engineered tendon nano‐constructs

**FIGURE 1:**
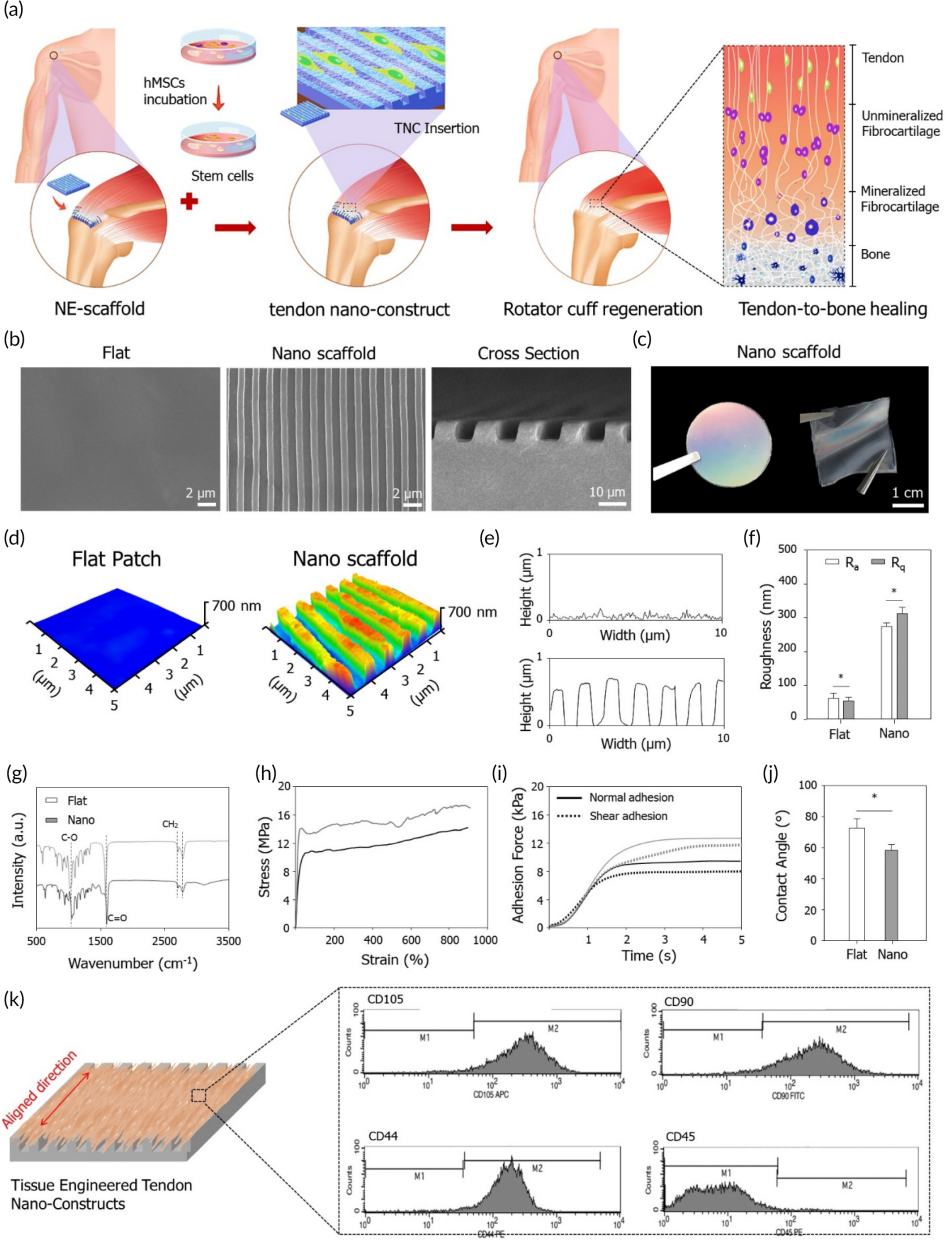
Rational design of the tissue‐engineered tendon nano‐constructs for rotator cuff (RC) tendon regeneration. (a) Schematic of tendon nano‐construct (TNC) therapy for repair of chronic rotator cuff tear (RCT). (b) Scanning electron microscopy (SEM) images of the fabricated the nano‐constructs. (c) Representative photograph of the flexible nano‐constructs. (d) AFM analysis of the nano‐constructs. (e) Roughness and (f) quantification of *R*_*a*_ and *R*_*q*_ values of the nano‐constructs. (g) Fourier transform‐infrared (FTIR) spectra of the nano‐constructs. (h) Strain–stress curves and (i) adhesion force of the nano‐constructs. (j) Water contact angels of the nano‐constructs. (k) Schematic of TNC and FACS analysis of the stem cells (*p < 0.05)

A schematic of the RCT repair strategy of TNC transplantation is shown in Figure 1a. Scanning electron microscopy (SEM) images of the surface morphologies of a flat scaffold and a tendon ECM‐inspired scaffold, as well as cross‐sectional SEM images, revealed the former construct to have a flat surface and the latter to have a highly aligned topography with grooves and ridges (~800 nm size) similar to those of the highly aligned structure of the native tendon ECM (Figure 1b,c). The 3D structures of these two scaffolds were confirmed using atomic force microscopy (Figure 1d). Their roughness values were as follows: arithmetic average roughness (*Ra*) = 54.12 nm and root‐mean‐squared roughness (*Rq*) = 48.77 nm for the flat scaffold; and (*Ra*) = 291.93 nm and (*Rq*) = 320.68 nm for the ECM‐inspired scaffold, indicating that the nanotopography had increased the surface roughness (Figure 1e,f). Fourier transform‐infrared (FTIR) spectroscopic analysis of the scaffold functional groups revealed characteristic absorption bands related to PCL (i.e., CH_2_ asymmetric stretching at 2940 cm^−1^, symmetric stretching at 2860 cm^−1^, C=O stretching vibration of carbonyl groups at 1720 cm^−1^, and C—O deformation at 1160 cm^−1^) in both scaffolds (Figure 1g). The mechanical properties of the scaffolds were measured using strain–stress and adhesion tests (both normal and shear adhesion forces). As previously reported by our research group, the tensile stress of the nanotopographic scaffold was greater than that of the flat scaffold, as was evident upon application of a load along the direction of the aligned nanostructure. Similarly, both normal and shear adhesion forces were higher in the nanotopographic scaffold (Figure 1h,i; detailed mechanical property results are shown in Table S1). An assessment of the scaffold wettability through measurement of the water contact angle showed that the value for the nanotopographic scaffold (58.17 ± 2°) was lower than that for the flat scaffold (78.3 ± 2°). In our previous report, we confirmed that the contact angle was larger in the direction of parallel to the nanogroove than in the direction of perpendicular to the nanogroove. In this work, the contact angle was measured parallel to the nanogroove (Figure 1j). The formation of aligned tendon tissue was observed on the nano scaffolds cultured hMSCs. To characterize the hMSCs used in this study, their expression of specific surface markers was analyzed using fluorescence‐activated cell sorting. The hMSCs at passage 3 showed positive CD105, CD90, and CD44 expressions and a negative CD45 expression (Figure 1k).

### 2.2 Tendon regeneration effect of tissue‐engineered tendon nano‐constructs in rabbit animal model

**FIGURE 2:**
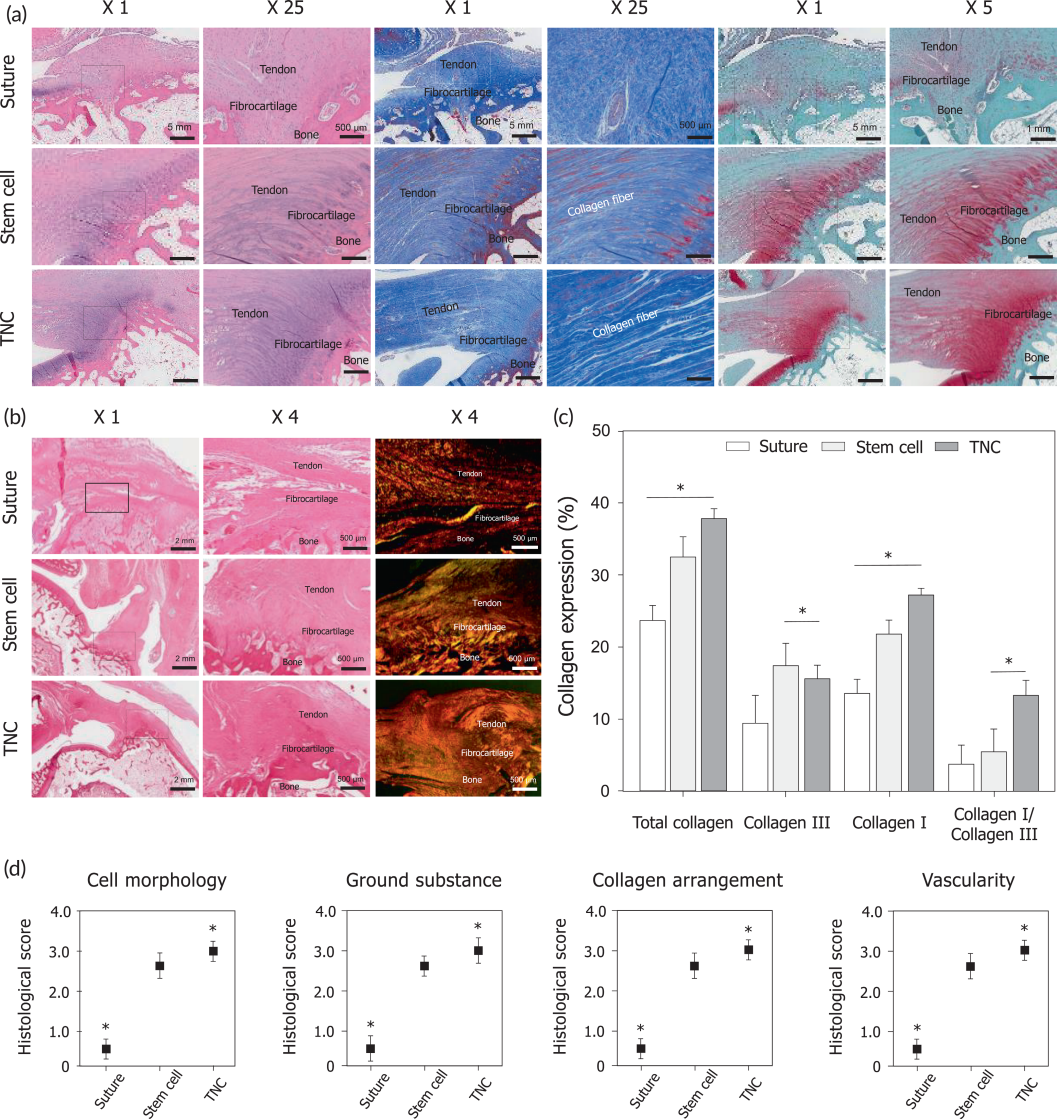
Effects of the tissue‐engineered tendon nano‐constructs on rotator cuff (RC) tendon regeneration in rabbit animal model. (a) Representative histological images of hematoxylin and eosin (H&E), Masson's trichrome (M&T), Safranin O staining of the supraspinatus tendon 6 weeks after repair, stem cell injection, and tendon nano‐construct (TNC) therapy. (b) Representative histological images of picrosirius red staining of the of the supraspinatus tendon 6 weeks after repair, stem cell injection, and TNC therapy and (c) quantification of collagen deposition (collagen I, collagen III, and ratio of collagen I and collagen III) through the image J software. (d) Histological score analysis on repaired RC tendon based on the Bonar scoring (*p < 0.05)

Our in vivo study demonstrated that the TNC could promote RC tendon regeneration, indicating that this construct contributes the following three clinically important advances in RCT repair: (i) the ability to evaluate RC tendon regeneration in large animal models, (ii) effective healing of the TBI junctions; and (iii) bone healing via the cascade effect. First, we hypothesized that the TNC with a highly aligned structure that mimics the native RC tendon would promote TBI regeneration (including the RC tendon, fibrocartilages, and bone). To prove our hypothesis, the TNC was tested in both rabbit and pig models of chronic RCT. We then compared the performance of three RCT therapies in this study: (i) the transosseous suture method (the current standard); (ii) stem cell therapy (a recent advanced method), conducted by injecting hMSCs mixed with fibrin gel into the injury site for 6 weeks; and (iii) transplantable TNC therapy (our new approach), conducted by grafting the nano‐construct (size: 3 cm × 3 cm) onto the defect for 6 weeks. The healing efficacy of each method was evaluated histologically, using hematoxylin and eosin (H&E) staining for viewing the tissue and cell morphology, Masson's trichrome (M&T) staining for determining the collagen arrangement, and Safranin‐O staining for observing fibrocartilage formation (Figure 2a). Interestingly, H&E and M&T staining revealed the collagen fibers of the regenerated tissue to be better organized, much more aligned, and much denser following TNC therapy than those resulting from stem cell therapy (Figure 2a). In contrast, the suture therapy resulted in a sparse collagen arrangement and tissue structure (Figure 2a). Furthermore, the fibrocartilaginous junctions in the TBI showed the highest degree of regeneration after TNC therapy when compared with those in the other therapy groups (Figure 2a). These results indicate the clinical significance of a precisely aligned nanotopography and the influence of stem cells in synergistically guiding the tendon tissue and promoting TBI regeneration. Picrosirius red staining was further performed to confirm the detailed formation and arrangement of collagen I and collagen III in the regenerated tissue. The TNC therapy not only resulted in the highest total collagen deposition but also significantly improved the percentage of collagen I, which provides for a dense tendon tissue structure with high mechanical strength (Figure 2b). Specifically, the rate of collagen I deposition was in the order of suture therapy (13%) < stem cell therapy (21%) < TNC therapy (26%) (Figure 2c). To quantify these histological results, semi‐quantitative scoring was performed using the Bonar scoring system (Figure 2d and Table S2). Both the TNC and stem cell therapy groups had significantly higher scores than that of the suture group, with the values in the TNC therapy group being the most statistically significantly.

### 2.3 Tendon regeneration effect of tissue‐engineered tendon nano‐constructs in large animal model

**FIGURE 3:**
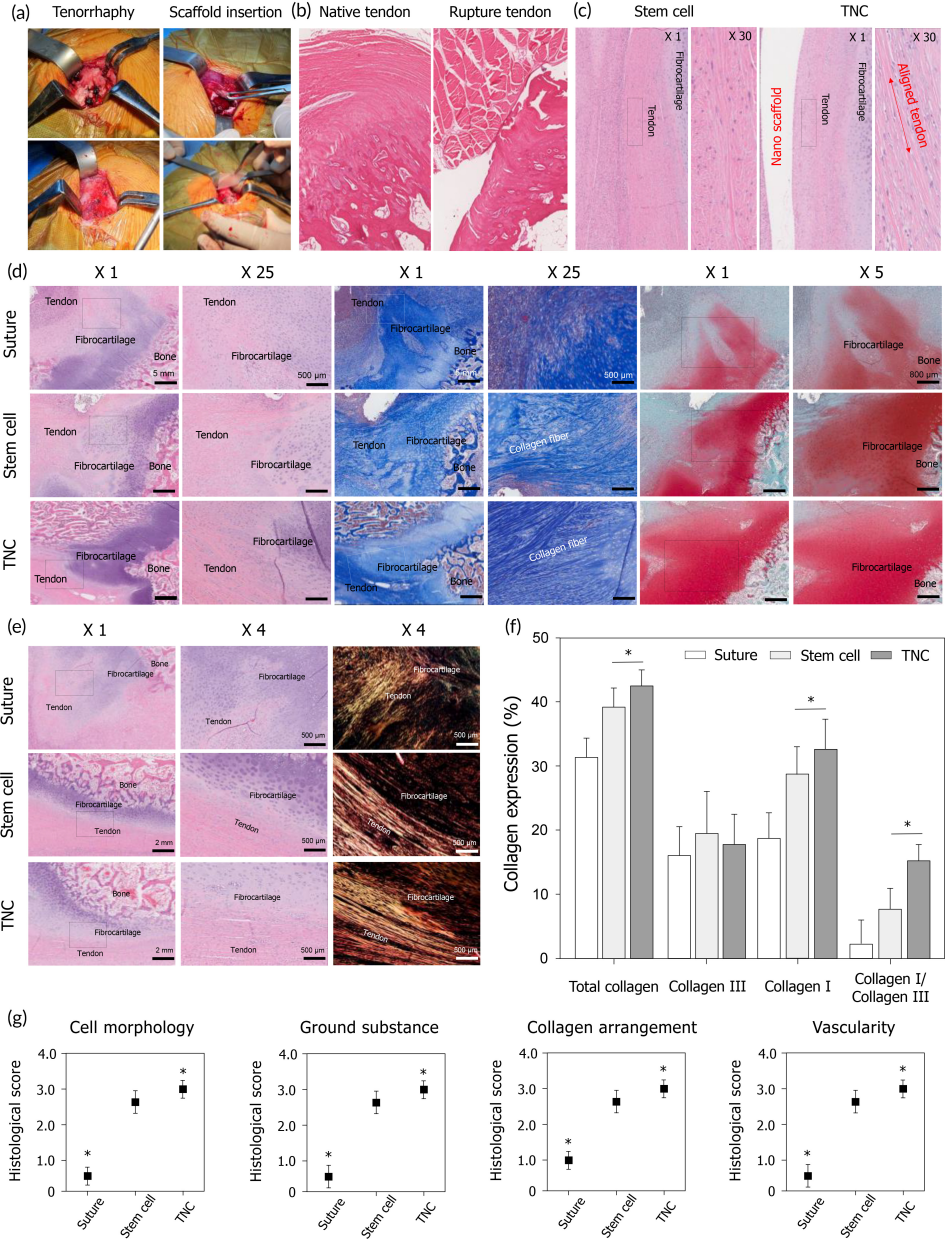
Effects of the tissue‐engineered tendon nano‐constructs on rotator cuff (RC) tendon regeneration in large animal model. (a) Surgical procedure of tendon nano‐construct (TNC) therapy for RC tendon repair and (b) histological images of rotator cuff tear after 4 weeks rupture. (c) The comparison histological images of repaired RC tendon between the stem cell injection and TNC therapy according to the presence of nano‐construct. (d) Representative histological images of hematoxylin and eosin (H&E), Masson's trichrome (M&T), and Safranin O staining of the supraspinatus tendon 6 weeks after repair, stem cell injection, and TNC therapy. (e) Representative histological images of picrosirius red staining of the of the supraspinatus tendon 6 weeks after repair, stem cell injection, and TNC therapy and (f) quantification of collagen deposition (collagen I, collagen III, and ratio of collagen I and collagen III) through the image J software. (g) Histological score analysis on repaired RC tendon based on the Bonar scoring (*p < 0.05)

To confirm the regenerative effect of the TNC in a larger animal, the entire evaluation was repeated in a pig model of RCT. Figure 3a depicts the patch transplantation process, and Figure 3b shows images of native and ruptured RC tendon tissues. Interestingly, the compact and aligned tendon tissue was formed below the TNC insertion compared with hMSCs only (Figure 3c). Importantly, the TNC therapy again resulted in the well‐organized, highly aligned, and highly dense collagen fibers to the greatest extent, as is evident in the H&E and M&T staining images (Figure 3d). In general, the safranin‐O‐staining is performed to confirm the mineralized fibrocartilage regeneration, we also confirmed the mineralized fibrocartilage regeneration by the staining. The staining images show a higher mineralized fibrocartilage regeneration in our TNC therapy compared to suture and stem cell therapy in both rabbit and pig model (Figures 2 and 3d). For the more accurate comparison of mineralized fibrocartilage regeneration, we have added the quantification data of stained area (Figure S2). Furthermore, to confirm the mineralization of hMSCs, we performed the osteogenic mineralization of hMSCs on the nano scaffolds. The quantification of the osteogenic mineralization on the flat and nano scaffolds demonstrated the highest degree of osteogenic mineralization by the hMSCs on the nano surface. In previous studies, the correlation between the osteogenic mineralization and fibrocartilage regeneration has been reported. These in vivo and in vitro results demonstrated that the proposed TNC therapy promotes the mineralized fibrocartilage regeneration. Also, these results again indicate that the precisely aligned nanotopography and stem cells could synergistically promote TBI regeneration not only in rabbits but also in pigs, whose RC tendon is known to be similar to that of humans. As in the rabbit study, Picrosirius red staining of the regenerated pig tissue was performed, whereupon the highest total collagen deposition along with a significantly improved collagen I percentage was shown in the TNC group (Figure 3e). The rate of collagen I deposition in the pig model was in the order of suture therapy (16%) < stem cell therapy (28%) < TNC therapy (32%) (Figure 3f). Bonar scoring of the histological results again showed stem cell therapy and TNC therapy to have significantly higher values than those of suture therapy (Figure 3g and Table S2). Therefore, these successful outcomes of TBI regeneration in both the rabbit and pig models of RCT suggest the clinical applicability of the proposed TNC.

### 2.4 Analysis of bone formation and biomechanical properties of regenerated tendon tissue

**FIGURE 4:**
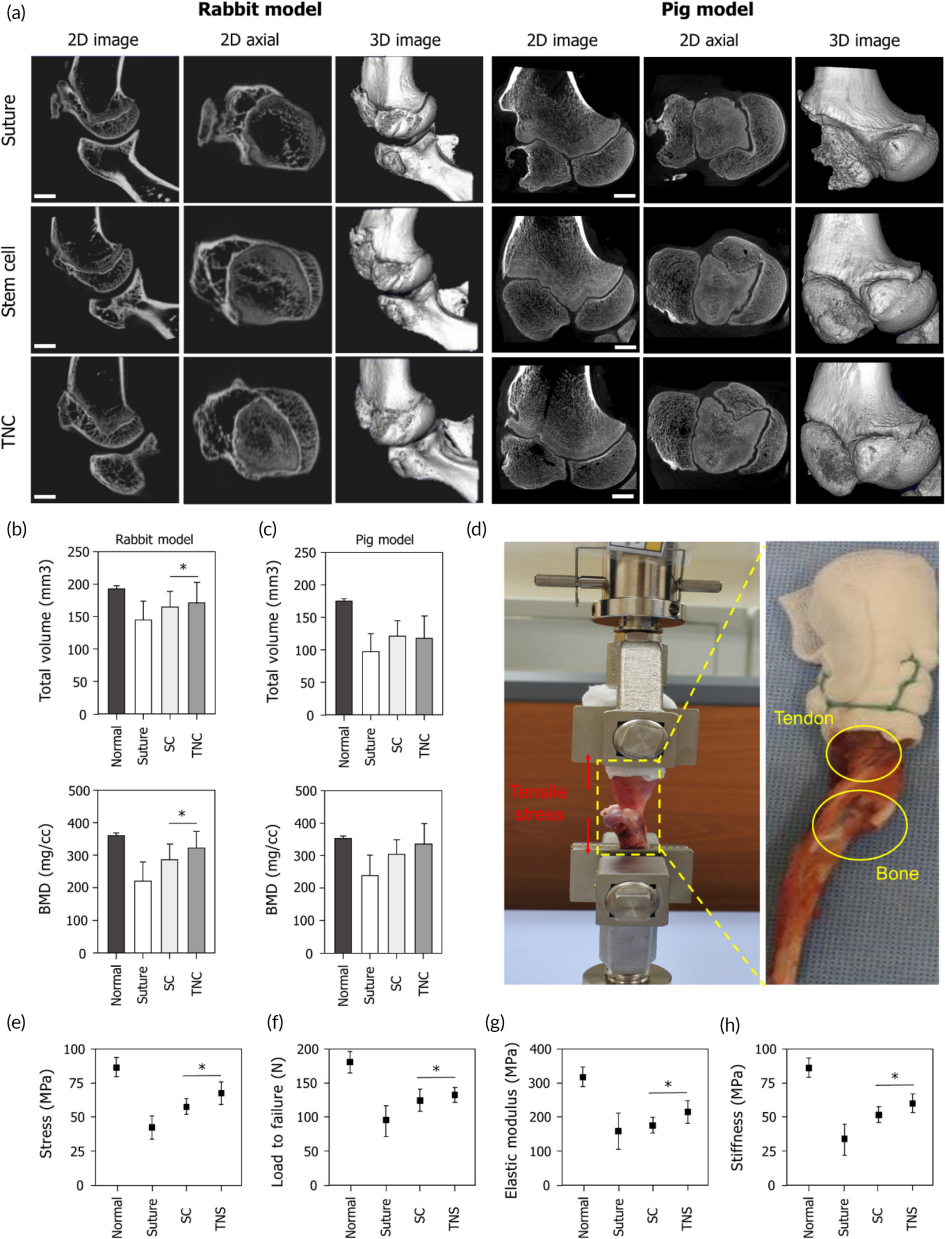
Effects of the tissue‐engineered tendon nano‐constructs on bone healing and biomechanical properties of rotator cuff (RC) tendon. (a) Representative micro‐CT images of the repaired bone tissues after repair, stem cell injection, and tendon nano‐construct (TNC) therapy in rabbit and pig rotator cuff tear (RCT) models. (b,c) Quantification of total volume and bone mineral density of repaired bone tissues. (d) Optical images of the biomechanical test of the repaired RC tendon. (e–h) Quantification of tensile stress, load to failure, elastic modulus, and stiffness of the repaired RC tendon (*p < 0.05)

In the evaluation of RCT repair techniques, the functional evaluation of the regenerated RC tissue is as important as histological evaluations. In this study, micro‐computed tomography (CT) and biomechanical tests were used for the functional evaluation of the regenerated RC tendon. Reconstructed micro‐CT images of the proximal humerus were used to determine the morphologies of the regenerated bone in both the rabbit and pig models (Figure 4a). The tendon–bone insertion site of the proximal humerus showed more significant bone formation after TNC implantation than after suturing or stem cell injection. The tissues repaired using the TNC showed increases in their total volume and bone mineralization density when compared with those repaired with the other methods in both animal models (Figure 4b,c). These results suggest that our TNC has a remarkable capacity for enhancing new bone formation at the TBI, with a potentially higher success rate in healing RCTs than that of currently used methods. Furthermore, at 6 weeks, the cross‐sectional area of the TNC‐repaired tendon tissue was higher (~221 mm^2^) than that of the stem cell‐repaired (~202 mm^2^) and suture‐repaired tendon tissues (~148 mm^2^) (Figure S1A) and the ultimate stress was higher (~78.8 MPa) than that of the stem cell‐repaired (~67.4 MPa) and suture‐repaired tendon tissues (~48.1 MPa) (Figure S1B). Also, tensile strength was higher (~74.2 MPa) than that of the stem cell‐repaired (~53.4 MPa) and suture‐repaired tendon tissues (~28.8 MPa) (Figure 4e). The ultimate failure loads were higher in the TNC therapy group (127 ± 3 N) than in the stem cell (118 ± 6 N) and suture therapy groups (86 ± 7 N) (Figure 4f), as were the Young's modulus values (~198 MPa in the TNC group vs. ~166 MPa in the stem cell group and ~148 MPa in the suture group) (Figure 4g). Finally, the tissue stiffness was also higher in the TNC therapy group (~61 MPa) than in the stem cell and suture therapy groups (~52 and ~41 MPa, respectively) (Figure 4h). These results indicate that the mechanical strength of the supraspinatus TBI had been successfully augmented with the TNC.

### 2.5 Cell morphological response to tissue‐engineered nano‐constructs for tendon regeneration

**FIGURE 5:**
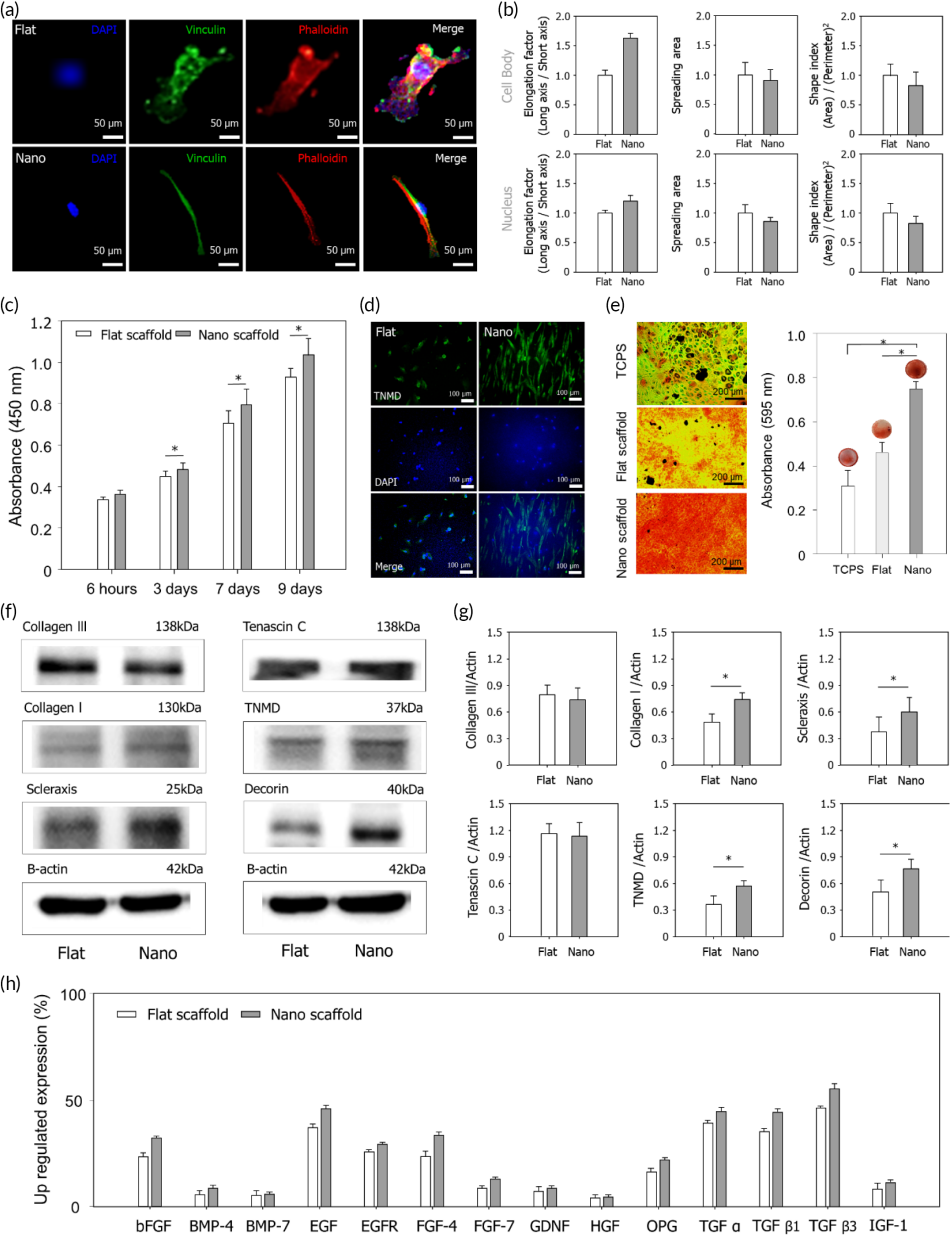
Effects of the tissue‐engineered nano‐constructs on cell morphology and functions for RC tendon regeneration. (a) Representative immunofluorescence images depicting DAPI (blue), vinculin (green), and phalloidin (red) staining of human mesenchymal stem cells (hMSCs) on the nano‐construct. (b) Quantification of the elongation factor, spreading area, and shape index of the cell body and nucleus of the hMSCs on the nano‐construct. (c) Cell attachment and proliferation of hMSCs on the nano‐constructs for 6 h, 3 days, 7 days, and 9 days. (d) Tenogenic differentiation of hMSCs on the nano‐constructs (green: TNMD, blue: DAPI). (e) Osteogenic mineralization of hMSCs on the nano‐constructs for 21 days. (f,g) Western blot analysis and quantification of collagen III, collagen I, scleraxis, tenascin C, TNMD, decorin protein expression in hMSCs on the nano‐constructs. (h) Expression of upregulated growth factors in hMSCs on the nano‐constructs (*p < 0.05)

As a possible mechanism underlying the TBI regeneration effect mediated by the TNC therapy, we propose that the healing process may occur through the following steps: (i) the host tissue cells and the hMSCs on the scaffold are elongated by the aligned nanostructures which is a similar structure to the ECM of tendon tissue in vivo, migrating the cells to the defect site; (ii) the hMSCs delivered by the scaffold may differentiate directly into tenocytes as well as promote the autocrine/paracrine effects, which may eventually induce the tendon regeneration; (iii) the elongated cell morphology induced by the nanotopographic cues could promote the differentiation and maturation of the hMSCs and surrounding cells; finally, (iv) regenerated highly aligned and well‐organized tendon tissue affected by the matured and differentiated cells induces overall healing of the TBI with a cascade healing effect. From this standpoint, our in vitro study demonstrated that the morphology of the stem cells was controlled, and the functions of these cells were improved by the tendon ECM‐like nanostructures. The origin of these improvement of cellular functions is high alignment and organization of the scaffolds which mimics the highly aligned collagen fibers and nanoscale ECM in the native tendon. It is well known that the cellular morphology and functions of stem cells are sensitively controlled by ECM‐like nanotopographic cues.^33^, ^34^, ^35^ Also, in our previous studies, we had also demonstrated that various nanostructures mimicking the ECM could control the morphology of stem cells and promote cell migration and differentiation.^19^, ^36^, ^37^, ^38^ With these findings as a guide, the hMSCs on the nanotopographic scaffold were analyzed using immunohistochemical staining to confirm the effects of nanotopographic cues on the cellular morphology (e.g., cell shape, focal adhesion, and orientation) at the single‐cell level. As shown in Figure 5a, the hMSCs had reacted sensitively to the nanotopographic highly aligned cytoskeletal structures on the scaffold, displaying a highly aligned shape and orientation along the aligned nanotopography (>50% elongated morphology). The stem cells also had different spreading patterns, showing a less spread cell morphology on the nanotopographic scaffold when compared with that on the flat one (<20% spreading morphology) (Figure 5b). The elongation factor (defined as the long‐axis/short‐axis ratio), area, and shape index (defined as the area/perimeter^2^ ratio) of both the nuclei and cell body were also estimated on the two types of scaffolds. The elongation factors of the cell bodies and nuclei were significantly increased by the nanotopographic cues, with the cell bodies being more sensitively affected (Figure 5b). Because of the differences in cell morphology between the flat and nanotopographic scaffolds, we hypothesized that the nanotopographic cues could be responsible for the enhanced cellular functions related to RC tendon regeneration.

### 2.6 Effects of tissue‐engineered nano‐constructs cues on stem cell functions

Although there was no significant difference in hMSCs attachment on the two types of scaffolds, cell proliferation was more than 10% higher on the ECM‐inspired scaffold after 3 days (Figure 5c). Additionally, we examined the osteogenic mineralization of hMSCs on the nanotopographic scaffolds. Alizarin Red S staining revealed higher calcium levels on the ECM‐inspired scaffold than on the flat scaffold and tissue culture polystyrene substrate (TCPS). To demonstrate the tenogenic differentiation of hMSCs, we have added TNMD staining data. The stained image and quantification results showed enhanced tenogenic differentiation of hMSCs on the nano‐construct compared to flat‐construct (Figure 5d and S5). Quantification of the osteogenic mineralization further confirmed that the highest degree of osteogenesis had occurred with the cells cultured on the ECM‐like scaffold (Figure 5e), suggesting that the nanotopographic environment, which was similar to the complex microenvironment of the tendon ECM, had aided in promoting the proliferation and mineralization of hMSCs. To verify whether the nanotopographic cues could affect the expression of proteins related to the induction of the dense and well‐organized RC tendon tissue and tenogenic differentiation of the hMSCs, western blot analysis was carried out to detect those specific proteins. Collagen type I, scleraxis, tenomodulin, and decorin, which play important roles in tenogenic differentiation and tendon regeneration, were found to be upregulated in the cells on the nanotopographic scaffold relative to their expression in cells on the flat scaffold. In contrast, collagen type III was slightly downregulated on the ECM‐inspired scaffold (Figure 5f,g). It is known that stem cells generate autocrine and paracrine factors, which are crucial molecules for signaling the activation of various cellular functions. The secretion of growth factors by hMSCs cultured on the two types of scaffold was analyzed. The hMSCs on the ECM‐inspired scaffold secreted higher levels of basic fibroblast growth factor, bone morphogenetic protein (BMP)‐4, BMP‐7, epidermal growth factor, epidermal growth factor receptor, fibroblast growth factor (FGF)‐4, FGF‐7, glia cell‐derived neurotrophic factor, hepatocyte growth factor, osteoprotegerin, transforming growth factor (TGF)‐alpha, TGFβ1, TGFβ3, and insulin‐like growth factor‐1 (Figure 5h).

In conclusion, our results have demonstrated that our rational design of a tissue‐engineered TNC has great potential for use in the repair of chronic RCTs, as it facilitated functional tendon regeneration in large animal models, the findings of which were supported by in vitro test results. We propose that this new TNC therapy could replace the current surgical methods used for RCT repair and improve shoulder functions through its healing of the TBI.

## 3 MATERIALS AND METHODS

### 3.1 Stem cell source

All human adipose‐derived mesenchymal stem cells (hMSCs) used in this study were purchased from SCIENCELLTM. The hMSCs were cultured in DMEM low‐glucose (Cellgro, USA) supplemented with 10% fetal bovine serum (FBS; Cellgro, USA) and 1% penicillin–streptomycin (GenDEPOT, Houston, TX, USA) at 37°C in a 5% CO_2_ atmosphere. The medium was changed every 3 days. All stem cells used in this work were at passage 3–5.

### 3.2 Design and fabrication of a nanoengineered‐artificial stem cell construct

The detailed method to fabricate PCL‐based tendon‐inspired scaffold has already been reported by our group. Briefly, a thin PCL scaffold was fabricated by spin coating the PCL solution that was poured into glass on the vacuum cuck of the spin coater. After the thermal imprinting process, the assembly of the PCL layer on circular glass and PDMS molds was cooled at 25°C for 30 min. The fabrication process of the tendon‐inspired scaffold is following which PDMS molds were used to create either a flat or a nanotopographical (800 nm, ridges, and grooves) pattern on the melted film surface under pressure at 80°C for 2 min.

### 3.3 Characteristics and properties analysis

The tissue‐engineered nano‐constructs were analyzed using high‐resolution field‐emission scanning electron microscope (FE‐SEM), FTIR spectroscopy. FE‐SEM images of the surface of all scaffolds fabricated in this study were observed using a JSM‐7500F microscope (Oxford, UK) at an acceleration voltage of 15.0 kV and average working distance of 8.8 mm. The samples were coated with platinum prior to morphological observation. Chemical characteristics of tissue‐engineered nano‐constructs were analyzed to confirm their chemical structures. The chemical bond structures were examined by FTIR (Spectrum 400, USA). The static water contact angle of liquids was measured using customized camera systems with a Computer M1214‐MP2 2/3″ Fixed Lens and analyzed using the ImageJ software. For each measurement, 10 μl of water was drop‐dispensed onto the surface over a time span of 1 min. The water contact angle was then measured as the tangent to the interface of the droplet on the scaffolds. Measurements were repeated at least five times for each sample and averaged. All experiments were performed at room temperature.

### 3.4 Mechanical properties analysis

The mechanical tests of the all scaffolds were performed using MCT‐1150 tensile testers (A&D Company, Japan) at a test speed of 100 mm/min. The tests included the analysis of 10 specimens per sample with the same interval set. Normal and shear adhesion forces of all scaffolds were evaluated using porcine rind and measured using an MCT‐1150 instrument at a test speed of 50 mm/min. Prior to the adhesion test, a fresh porcine rind was rinsed with deionized water, all scaffolds were attached to its surface, and rind/scaffolds measured under a preload of ~0.5 cm^2^. The pulling weight was gradually increased until the adhesion force felled off.

### 3.5 hMSCs proliferation and mineralization analysis

hMSCs (1 × 10^4^ cells/samples) were seeded onto the samples and cultured for 6 h, 3 days, 7 days, and 9 days (cell proliferation), in DMEM. Quantitative analysis of cell proliferation on the samples was performed using a WST‐1 assay (Premix WST‐1 Cell Proliferation Assay System, Takara Bio Inc., Kusatsu, Japan). hMSCs (4 × 10^4^ cells/sample) were cultured for 14 days on samples in osteogenic differentiation medium. Alizarin Red S (Sigma‐Aldrich, USA) staining was used to confirm the osteogenic differentiation (according to the degree of mineralization) of hMSCs on sample surfaces. The stained cells were destained with cetylpyridinium chloride (Sigma‐Aldrich), and the extracted stains were measured using an absorbance reader (iMarkTM Microplate Absorbance Reader, Bio‐Rad, Hercules, CA, USA) at 595 nm to quantify the osteogenic differentiation of hMSCs.

### 3.6 Western blotting

To confirm the protein expression levels of collagen III, collagen I, scleraxis, tenascin C, TNMD, and decorin, hMSCs were first cultured on the tendon inspired scaffolds for 12 h, following which 1 × 105 cells/sample were cultured in medium (Promo Cell) for 7 days. Thereafter, the cells were washed twice with cold PBS and lysed by ultrasonication in RIPA buffer for 30 min at 4°C. The lysates were collected by centrifugation at 12,000 × g for 15 min at 4°C. After washing, the cells twice with cold PBS, they were lysed again with a modified radioimmunoprecipitation assay (RIPA) buffer (150 mM sodium chloride, 1% Triton X‐100, 0.5% sodium deoxycholate, 0.1% sodium dodecyl sulfate [SDS], 50 mM Tris [pH 8.0], 1 mM phenylmethylsulfonyl fluoride, 2 μg/ml leupeptin, 1 μg/ml pepstatin, 1 mM sodium orthovanadate, and 100 mM sodium fluoride) for 30 min at 4°C. The lysates were cleared by centrifugation at 14,000 × g for 15 min at 4°C. The protein content of the cell lysates was determined using the Micro BCA Assay Kit (Pierce, Rockford, IL, USA) according to the manufacturer's instructions.

### 3.7 Growth factors array

To ascertain whether the hMSCs could secrete growth factors and cytokines on the flat and nanotopographical scaffolds, the hMSCs (3 × 10^4^ cells/samples) were seeded on the scaffolds and cultured for 5 days in the proliferation medium. The hMSCs were then cultured for 3 days in the medium. Finally, the hMSCs were again cultured for 1 day in DMEM using the RayBio G‐Series Human Growth Factor Array 1 Kit (RayBiotech, Norcross, GA) according to the manufacturer's protocol.

### 3.8 In vivo animal study

The animal study was approved by the Ethics Committee of Chonnam National University Medical School and Chonnam National University Hospital. New zealand white rabbits (male) weighing 2.5–3.5 kg per group were equally divided into the suture (current therapy) group, stem cell injection group, and nanoengineered‐stem cell construct group. All rabbits were fully anesthetized with an intramuscular injection of 350 mg/kg ketamine (Youhan Corporation, Seoul, Korea) and 50 mg/kg xylazine hydro‐chloride (Rompun; Bayer HealthCare, Korea). Both shoulders of each rabbit were shaved and disinfected with povidone‐iodine (Firson, Korea), and the animals were placed in lateral position with the forelimbs in adduction and external rotation. A 3.0 cm skin incision over the scapulohumeral joint was made, subcutaneous tissue was dissected, and omotransverse and trapezius muscles were retracted to expose supraspinatus tendon (located in a superior position to the scapular spine). A sharp release of the supraspinatus tendon at a greater tuberosity of the humerus over a 10 mm width was observed for the acute RC tear model, and a surgical RC repair was performed immediately after the supraspinatus tendon tear. All rabbits (male) were fully anesthetized with an intramuscular injection of 35 mg/kg ketamine (Youhan Corporation) and 5 mg/kg xylazine hydrochloride (Rompun; Bayer HealthCare). Both shoulders of each rabbit were shaved and disinfected with povidone‐iodine (Firson, Korea), and the animals were placed in lateral position with the forelimbs in adduction and external rotation. A 2.0‐cm skin incision was made over the scapulohumeral joint, subcutaneous tissues were dissected, and the omotransverse and trapezius muscles were retracted to expose the supraspinatus tendon (located superior to the scapular spine). We used the right shoulder for suture group (*n* = 4) and the left shoulder for stem cell injection group (*n* = 4). For chronic RC tear model, we wrapped the detached tendon stump with silicone Penrose drain with 10 mm long and 8 mm outer diameter (Yushin Corp, Bucheon, Korea) to prevent adhesion to the surrounding soft tissue for 4 weeks until the secondary procedures could be performed. We used the left shoulder as nanoengineered‐stem cell construct group (*n* = 4) and the native tendon group (*n* = 4). The fascia and subcutaneous tissues were sutured using interrupted 3−0 vicryl sutures (Ethicon, Johnson & Johnson), and the skin was sutured with interrupted 4–0 prolene sutures. All rabbits and pigs (male) tolerated this procedure without any intraoperative complications. The rabbits and pigs were sacrificed 6 weeks after surgery to obtain tissues including the tendon, fibrocartilage, and bone regions of the rotator cuff.

### 3.9 Histological observations and evaluation

The proximal humerus including the greater tuberosity head with attached supraspinatus tendon of both shoulder of each rabbit was harvested. Specimens were fixed in neutral buffered 10% formalin (pH 7.4) and decalcified with Calci‐Clear Rapid (National Diagnostics, Atlanta) for 2 weeks, and paraffin blocks were made in the repair site including supraspinatus tendon and greater tuberosity. Sections (4 μm thickness) were cut in the coronal plane and stained with hematoxylin and eosin (H&E) and Masson's trichrome. We assessed cellularity, collagen fiber continuity, orientation, density, and maturation of the tendon‐to‐bone interface, and we also evaluated the inflammation rate around patch at the tendon‐to‐patch interface. Images were captured and acquired using an Aperio Image Scope (Leica, Ca, USA) software. General histological evaluation was performed with hematoxylin and eosin (200× magnification), Masson's trichrome (200× magnification), and Picrosirius red (100× magnification) stained slides of chronic RC tear animal models. The slides were evaluated using the semiquantitative grading scale of Bonar score, which assess four variables (cell morphology, ground substance, collagen arrangement, and vascularity) of tendon to bone interfaces. A four‐point scoring system is used, where 0 indicates a normal appearance and 3 a markedly abnormal appearance (Table S1). The total histological scores for each group were calculated from the sum of these four characteristic grades. Four sections were randomly selected form each group and were evaluated blindly by three independent assessors. The average score was used for comparison.

### 3.10 Analysis of RC tendon healing by microcomputed topography and biomechanical test

For micro computed topography (micro‐CT) evaluation (*n* = 4 and 2), humeral head of specimens with supraspinatus tendon were assessed using a Micro‐CT with a 59 μm isotropic voxel resolution under 90 kV voltage (Quantum FX, PerkinElmer, USA). After three‐dimensional reconstructed images were obtained, a customized 3.5‐mm × 3.5‐mm cylindrical region of interest (ROI) containing the distal portion of the supraspinatus tendon and the bony footprint was identified at the surface of the repaired supraspinatus tendon‐bone footprint according to postmortem observations. The bone volume fraction (bone volume; BV), bone mineral density (BMD) was calculated for a ROI located at the greater tuberosity. At 6 weeks after surgery, 18 animals were sacrificed for biomechanical testing (four rabbits per group per time point). The humerus with attached supraspinatus tendon was dissected from the surrounding tissues. For the biomechanical testing, the harvested tissues were wrapped in saline‐soaked gauze and kept at −80°C. Before testing, the tissues were thawed with saline wet gauze at room temperature for 24 h and the tissues were kept moist with saline during all of the tests. The proximal end of the tendon was compressed with sandpaper, gauge, and rubber to prevent slippage and to reduce damage to the specimens. The complex was clamped vertically in the custom‐designed upper jig. Testing was performed with the shoulders at 90° of abduction with a material testing system (H5K5; Tinus Olsen, England, UK). All specimens were initially preloaded to 0.2 N and preconditioned for five cycles under 5% of strain at a rate of 0.1 mm/s. Then, these specimens were loaded to failure in tension at a constant rate of 0.1 mm/s. The cross‐sectional area of the supraspinatus tendon was measured at the TBI. The load–displacement curve recorded during tests and the tensile stress, load to failure, ultimate stress, stiffness, and elastic modulus were calculated. In detail, Young's modulus is calculated following formula: Young's modulus = stress/strain and stiffness are calculated following formula: stiffness = Young's modulus * area/length.

## AUTHOR CONTRIBUTIONS

**Jangho Kim** and **Myung‐Sun Kim** supervised the project and final approval. **Yonghyun Gwon**, **Woochan Kim**, **Yang‐Kyung Kim**, **Sunho Park**, **Hyoseong Kim**, **Myung‐Sun Kim**, and **Jangho Kim** performed substantial contributions to conception and design, acquisition of the data or analysis and interpretation of the data. **Yonghyun Gwon** carried out the experiments. **Yonghyun Gwon**, **Woochan Kim**, **Yang‐Kyung Kim**, and **Sunho Park** analyzed the results on the experiments. **Woochan Kim**, **Yonghyun Gwon**, **Yang‐Kyung Kim**, **Sunho Park**, **Myung‐Sun Kim**, and **Jangho Kim** discussed the results and data in detail. **Yonghyun Gwon**, **Myung‐Sun Kim**, and **Jangho Kim** wrote the manuscript.

## CONFLICT OF INTEREST

Jangho Kim is named on patents that describe the use of stem cells platforms for tissue regeneration. All other authors declare that they have no competing interests.

### PEER REVIEW

The peer review history for this article is available at https://publons.com/publon/10.1002/btm2.10376.

## Supporting information


**Figure S1** Histological evaluation grade (Bonar score) to assess cell morphology, ground substance, collagen arrangement, and vascularity of repaired tendon to bone interface.
**Figure S2**. Quantification of fibrocartilage area determined by metachromasia with safranin O – staining
**Figure S3.** Histological evaluation grade (Bonar score) to assess cell morphology, ground substance, collagen arrangement, and vascularity of repaired tendon to bone interface.
**Figure S4**. Quantification of (A) cross‐sectional area and (B) ultimate stress of the repaired RC tendon.
**Figure S5**. Tenogenic differentiation intensity of TNMD.
**Table S1**. Histological evaluation grade (Bonar score) to assess cell morphology, ground substance, collagen arrangement, and vascularity of repaired tendon to bone interface.
**Table S2**. Distribution of histologic scores on repaired tendon to bone interface of chronic RC tear animal models using histological evaluation grades (Bonar score).

## Data Availability

All data needed to evaluate the conclusions in the paper are present in the paper and/or the supplementary materials. Additional data related to this paper may be requested from the corresponding author on reasonable request.
