# Supplementary material for: Tissue‐engineered tendon nano‐constructs for repair of chronic rotator cuff tears in large animal models
Source: Bioeng Transl Med. 2022 Jul 22;8(1):e10376. doi: 10.1002/btm2.10376 (PMC9842040; doi:10.1002/btm2.10376)
Supplement: Supplementary file 1 — Figure S1 Histological evaluation grade (Bonar score) to assess cell morphology, ground substance, collagen arrangement, and vascularity of repaired tendon to bone interface. Figure S2. Quantification of fibrocartilage area determined by metachromasia with safranin O – staining Figure S3. Histological evaluation grade (Bonar score) to assess cell morphology, ground substance, collagen arrangement, and vascularity of repaired tendon to bone interface. Figure S4. Quantification of (A) cross‐sectional area and (B) ultimate stress of the repaired RC tendon. Figure S5. Tenogenic differentiation intensity of TNMD. Table S1. Histological evaluation grade (Bonar score) to assess cell morphology, ground substance, collagen arrangement, and vascularity of repaired tendon to bone interface. Table S2. Distribution of histologic scores on repaired tendon to bone interface of chronic RC tear animal models using histological evaluation grades (Bonar score). [file BTM2-8-e10376-s001.docx]

Supporting information

**Tissue Engineered Tendon Nano-Constructs for Repair of Chronic Rotator Cuff Tears in Large-Animal Models**

Yonghyun Gwon^a,b,c^, Woochan Kim,^a,b,c^, Sunho Park^a,b,c^, Yang-Kyung Kim^c^, Hyoseong Kim^a,b,c^ , Myung-Sun Kim^d^*****, Jangho Kim^a,b,c^*****

^a^ Department of Convergence Biosystems Engineering, Chonnam National University, Gwangju 61186, Republic of Korea.

^b^Department of Rural and Biosystems Engineering, Chonnam National University, Gwangju 61186, Republic of Korea.

^c^Interdisciplinary Program in IT-Bio Convergence System, Chonnam National University, Gwangju 61186, Republic of Korea.

^d^Department of Physical and Rehabilitation Medicine, Chonnam National University Medical School & Hospital, Gwangju 61649, Republic of Korea.

**^*^**Correspondence should be addressed to J.K. ([rain2000@jnu.ac.kr](mailto:rain2000@jnu.ac.kr)) or M.K. ([mskim@chonnam.ac.kr](mailto:mskim@chonnam.ac.kr))


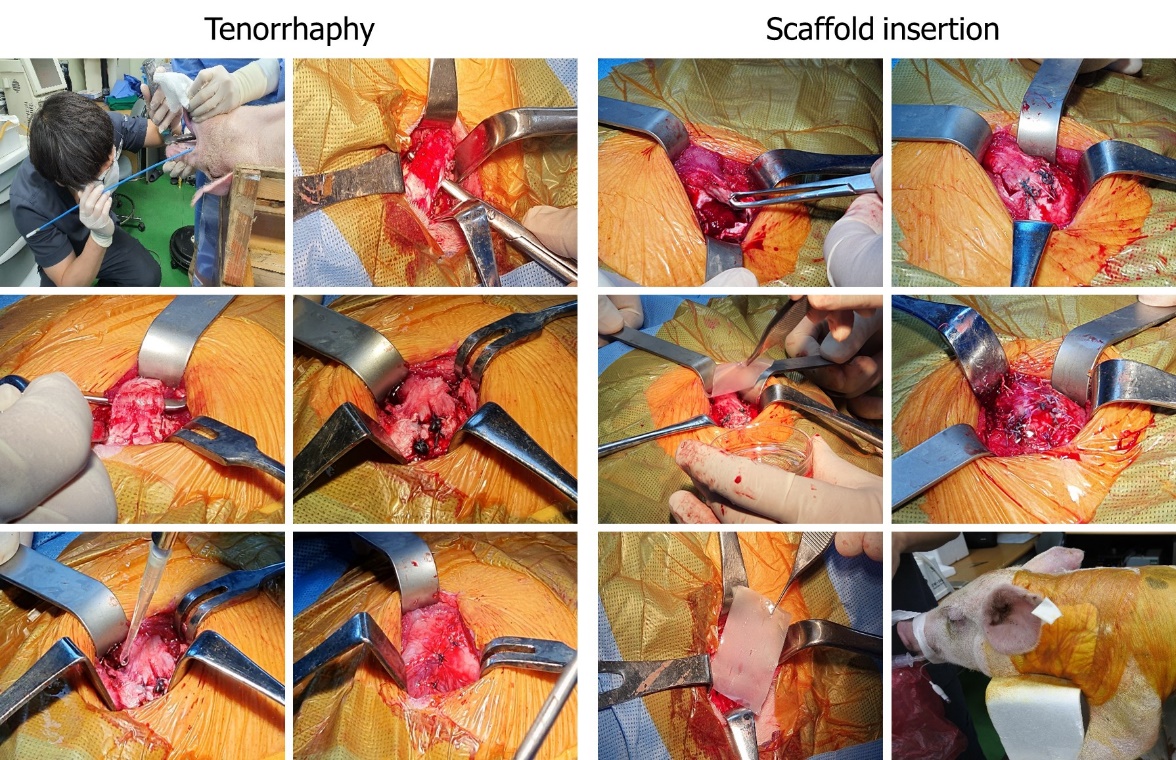


Figure S1. Histological evaluation grade (Bonar score) to assess cell morphology, ground substance, collagen arrangement, and vascularity of repaired tendon to bone interface.


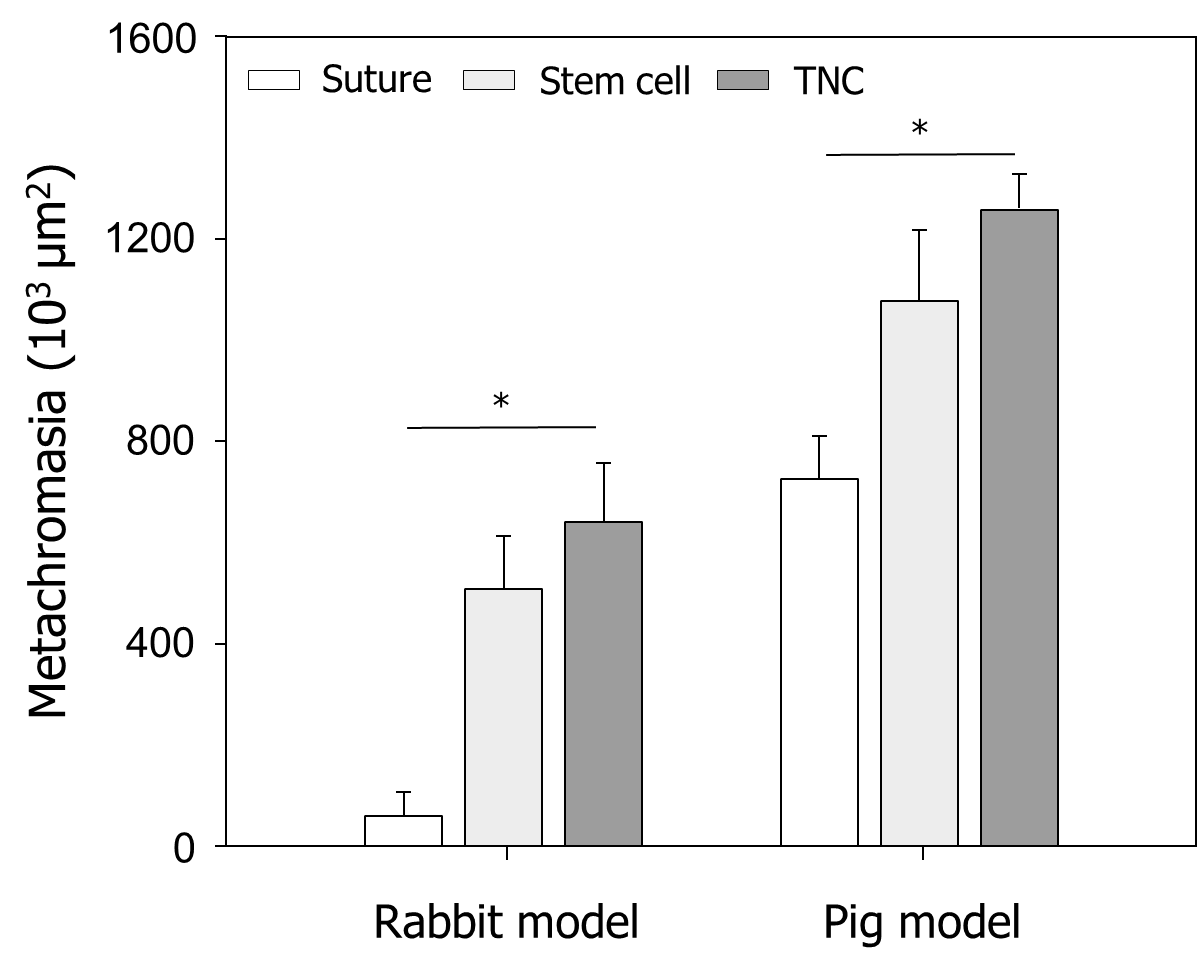


Figure S2. Quantification of fibrocartilage area determined by metachromasia with safranin O – staining


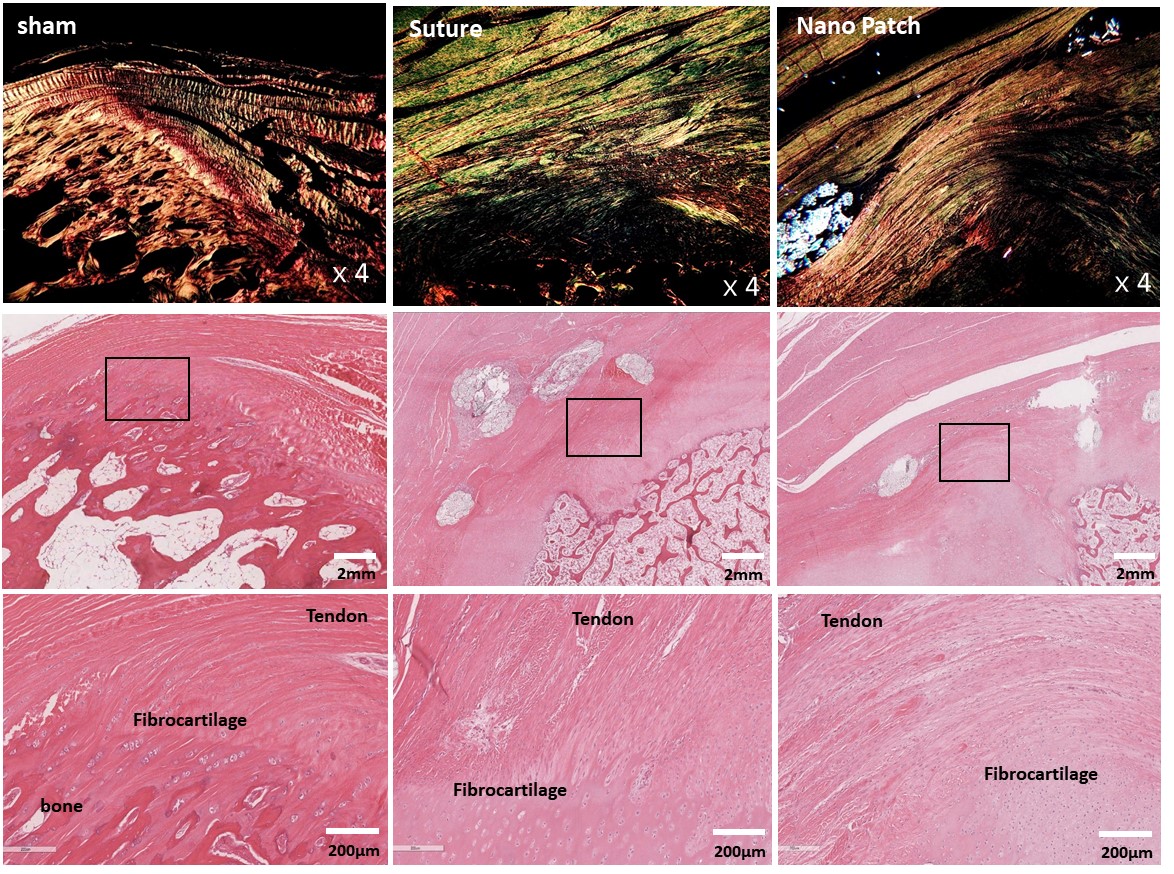


Figure S3. Histological evaluation grade (Bonar score) to assess cell morphology, ground substance, collagen arrangement, and vascularity of repaired tendon to bone interface.


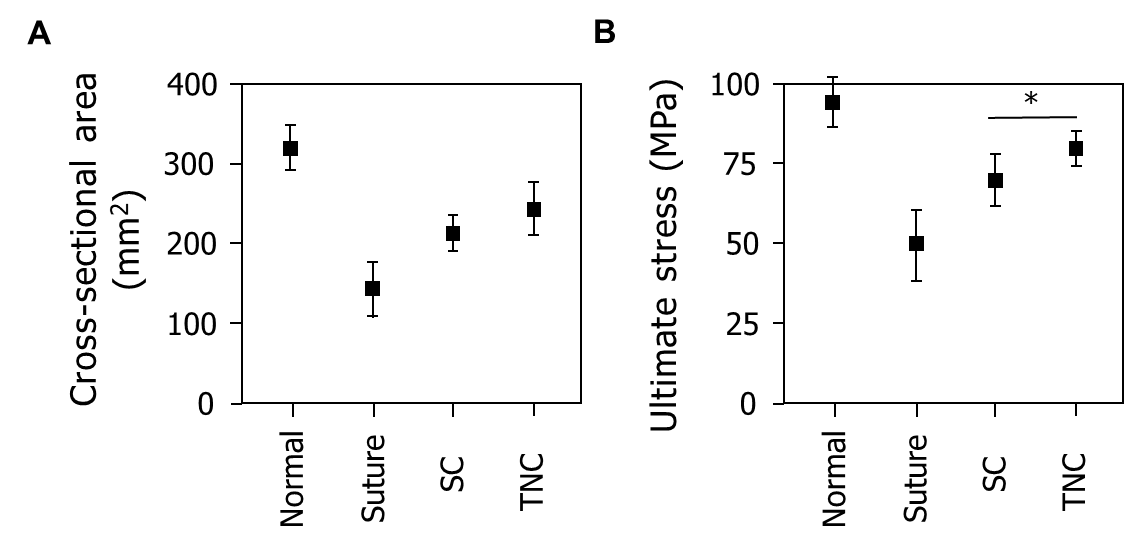


Figure S4. Quantification of (A) cross-sectional area and (B) ultimate stress of the repaired RC tendon.


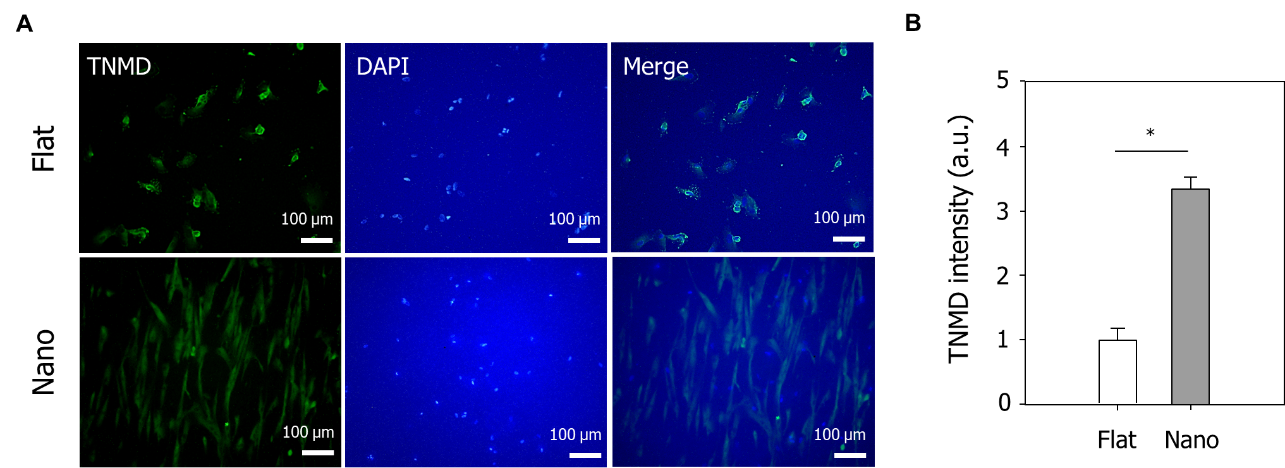


Figure S5. Tenogenic differentiation intensity of TNMD.


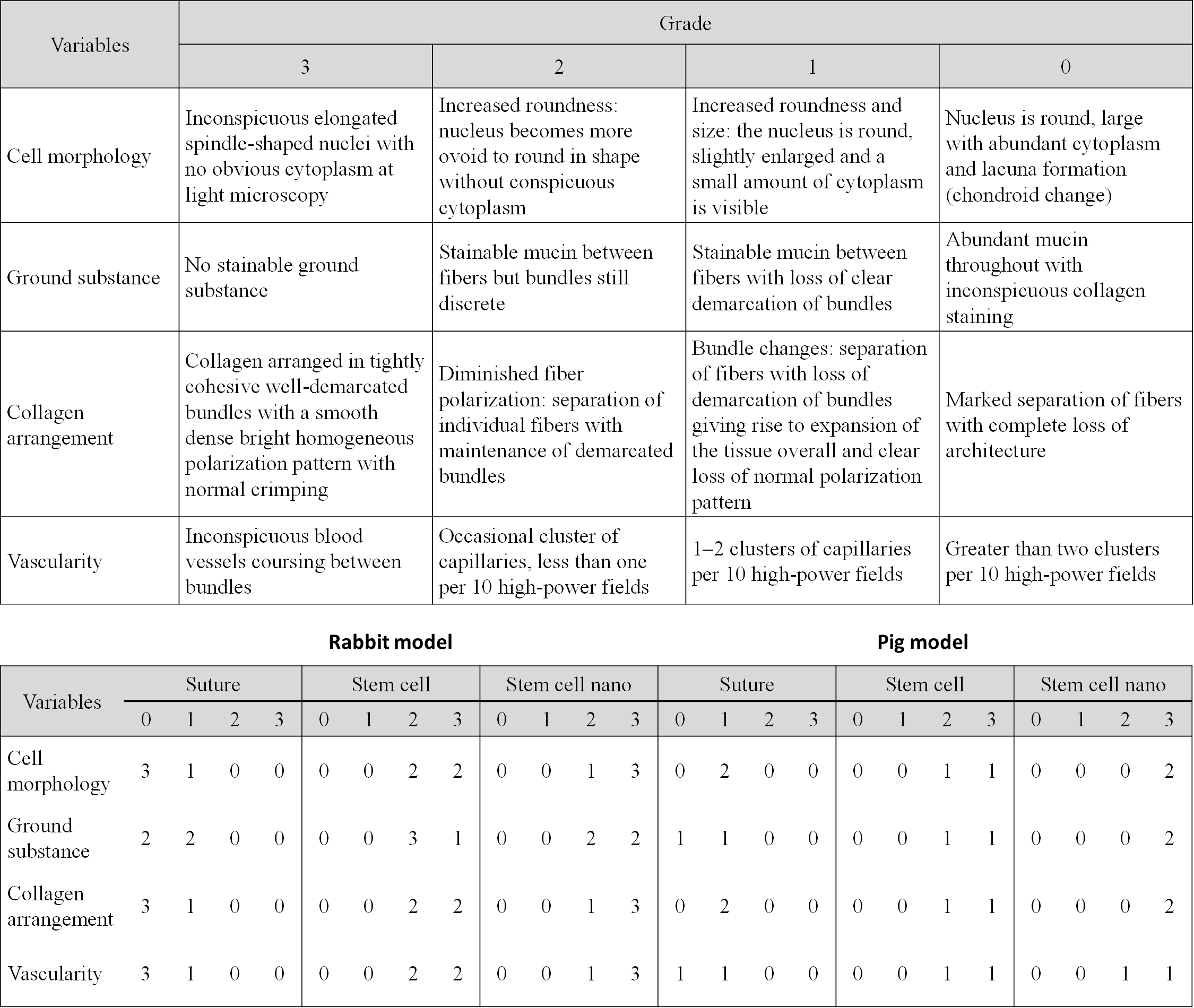


Table S1. Histological evaluation grade (Bonar score) to assess cell morphology, ground substance, collagen arrangement, and vascularity of repaired tendon to bone interface.


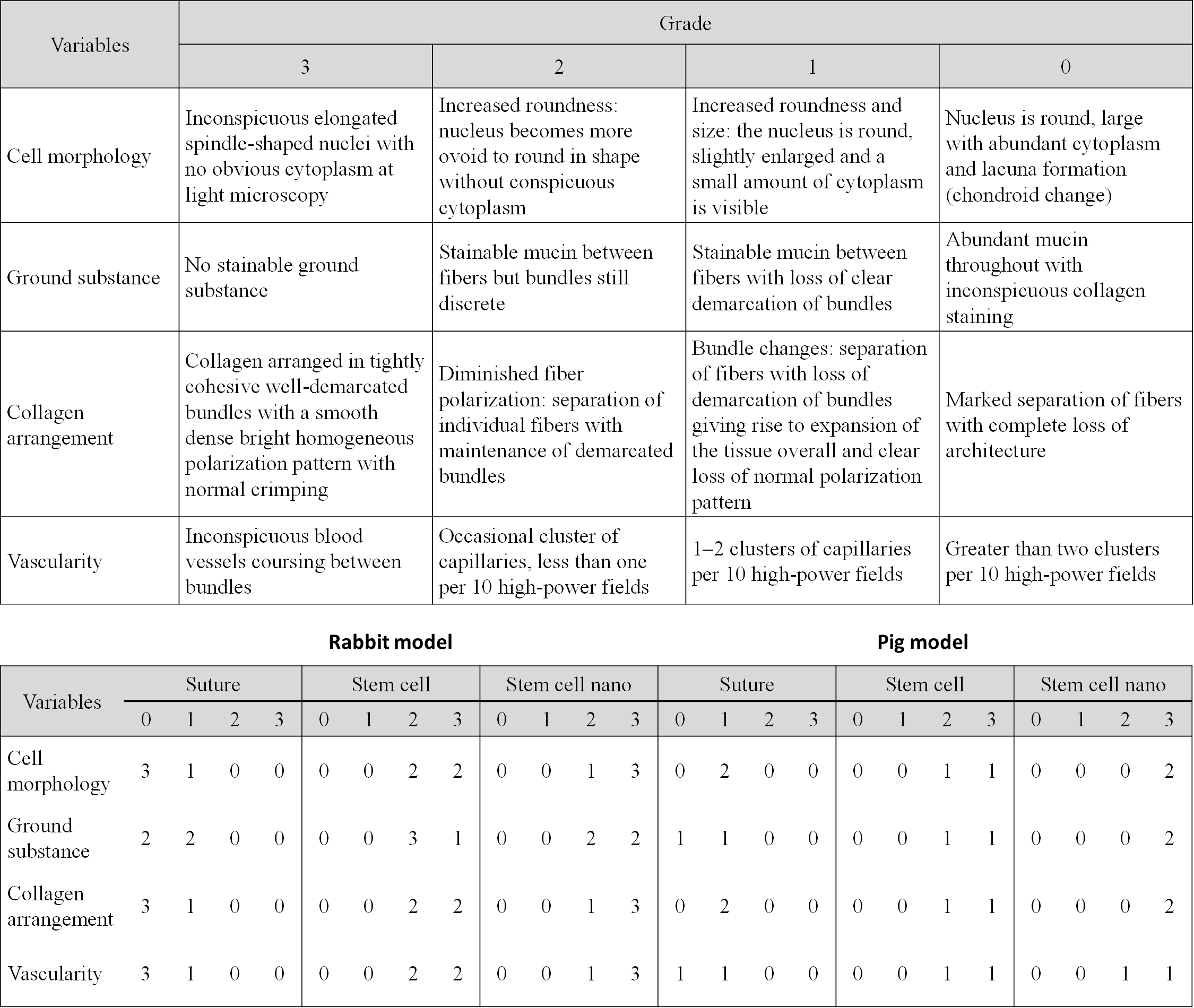


Table S2. Distribution of histologic scores on repaired tendon to bone interface of chronic RC tear animal models using histological evaluation grades (Bonar score).
